# SILVA, RDP, Greengenes, NCBI and OTT — how do these taxonomies compare?

**DOI:** 10.1186/s12864-017-3501-4

**Published:** 2017-03-14

**Authors:** Monika Balvočiūtė, Daniel H. Huson

**Affiliations:** University of Tübingen, Department of Computer Science, Sand 14, Tübingen, 72076 Germany

**Keywords:** Metagenomics, Taxonomic classification, OTU assignment, NCBI, Silva, RDP, Greengenes, Open tree of life

## Abstract

**Background:**

A key step in microbiome sequencing analysis is read assignment to taxonomic units. This is often performed using one of four taxonomic classifications, namely SILVA, RDP, Greengenes or NCBI. It is unclear how similar these are and how to compare analysis results that are based on different taxonomies.

**Results:**

We provide a method and software for mapping taxonomic entities from one taxonomy onto another. We use it to compare the four taxonomies and the Open Tree of life Taxonomy (OTT).

**Conclusions:**

While we find that SILVA, RDP and Greengenes map well into NCBI, and all four map well into the OTT, mapping the two larger taxonomies on to the smaller ones is problematic.

**Electronic supplementary material:**

The online version of this article (doi:10.1186/s12864-017-3501-4) contains supplementary material, which is available to authorized users.

## Background

**Fig. 1:**
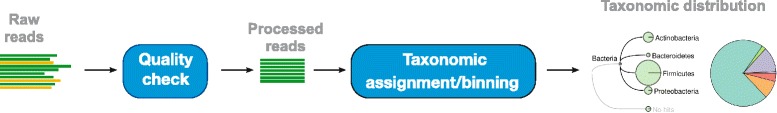
Basic taxonomic binning workflow

Microbiome sequencing analysis is concerned with sequencing DNA from microorganisms living in certain environments without cultivating them in laboratory. In a typical taxonomy guided approach [1], sequencing reads are first binned into taxonomic units and then the microbial composition of samples is analyzed and compared in detail (see Fig. 1).

The two main technical ingredients of taxonomic analysis are the reference taxonomy used and the binning approach employed. Binning is usually performed either by aligning reads against reference sequences (e.g. [2]) or using k-mer based techniques (e.g. [3]). Taxonomic binning of 16S reads is usually based on one of these four taxonomies: SILVA [4], RDP [5], Greengenes [6] or NCBI [7].

How important is the choice of reference taxonomy, given the known inconsistencies of microbial classifications [8]? To address this, the aim of this paper is to determine how similar these four taxonomies are, and whether results obtained using one classification can easily be carried over to another.

We define and explore an algorithm for mapping one taxonomy into another. This method allows us to compare taxonomies and is the basis for a tool that makes analyses on different classifications comparable to each other by mapping them onto a common taxonomy. While our main focus is on the four most popular taxonomic trees, we also consider the recently published Open Tree of life Taxonomy (OTT) [9].

We found that SILVA, RDP and Greengenes can be mapped into NCBI and OTT with few conflicts, but not vice versa. There is a great deal of difference between taxonomies that arise because of the differences in size and structure.

### Taxonomic classifications

**Fig. 2:**
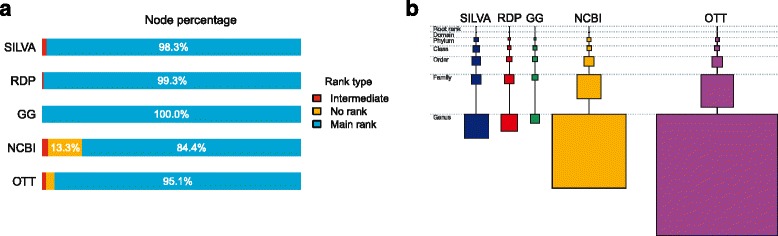
Composition of the five taxonomies. **a** Composition by rank type. *Main rank* stands for either root, domain, phylum, class, order, family, genus or species; *intermediate* includes all ‘sub–’, ‘infra–’, ‘super–’ etc. ranks. **b** Composition with respect to the number of nodes at each rank from root to genus. *Square areas* correspond to the number of nodes at each rank in each taxonomic classification

Each of the five taxonomies that we compare is based on a mixture of sources that have been compiled into taxonomies in different ways. They differ in both size and resolution (see Table 1). All taxonomies assign ranks to their nodes, the seven main ones being domain, phylum, class, order, family, genus and species. However, RDP and SILVA only go down to the genus level, whereas NCBI and OTT go down to the species level and below. The two latter taxonomies also have a number of intermediate ranks and contain many intermediate nodes (Fig. 2 a). To simplify the comparison of taxonomies, we will consider only nodes associated with the seven main ranks.

**Table 1:** Overview of five taxonomic classifications

Taxonomy	Type	No. of nodes	Lowest rank	Latest release
SILVA	Manual	12,117	Genus	Sep 2016
RDP	Semi	6,128	Genus	Sep 2016
Greengenes	Automatic	3,093	Species	May 2013
NCBI	Manual	1,522,150	Species	Today^a^
OTT	Automatic	2,627,066	Species	Sep 2016

^a^For the analyses we have used NCBI taxonomy as published on 5th Oct 2016

Figure 2 a shows the percentage of nodes that are assigned to a main rank in each of the five taxonomies. We found that all taxonomies have 1–2% of nodes with an intermediate rank (‘sub–’, ‘super–’ and other), except for Greengenes. Nodes with no rank assignment are found only in OTT (3.3%) and NCBI (13.3%). The latter taxonomic classification has the lowest percentage (84.4%) of nodes that fall into the category of main ranks.

Figure 2 b shows the composition of the five taxonomies at all ranks down to the level of genus. The NCBI taxonomy has 2.7 times fewer genera and 1.9 times fewer species (not shown) than the OTT. In the following we describe each of the five taxonomies in more detail (summarized in Table 1).

#### SILVA

The SILVA database [4] contains taxonomic information for the domains of Bacteria, Archaea and Eukarya. It is based primarily on phylogenies for small subunit rRNAs (16S for prokaryotes and 18S for Eukarya). Taxonomic rank information for Archaea and Bacteria is obtained from *Bergey’s Taxonomic Outlines* [10–13] and from the *List of Prokaryotic Names with Standing in Nomenclature* (LPSN) [14], whereas eukaryotic taxonomy is based on the consensus views of the *International Society of Protistologists* [15, 16]. Taxonomic rank assignments in the SILVA database are manually curated [4]. For the comparisons we used the taxonomy associated with SILVA small subunit ribosomal RNAs (16S/18S) v128 as released on 29/09/2016.

#### Ribosomal database project (RDP)

The RDP database [3] is based on 16S rRNA sequences from Bacteria, Archaea and Fungi (Eukarya). It contains 16S rRNA sequences available from the *International Nucleotide Sequence Database Collaboration* (INSDC) [17] databases. Names of the organisms associated with the sequences are obtained as the most recently published synonym from *Bacterial Nomenclature Up-to-Date* [18]. Information on taxonomic classification for Bacteria and Archaea is based on the taxonomic roadmaps by *Bergey’s Trust* [19] and LPSN [14]. Taxonomic information for fungi is obtained from a hand-made classification dedicated to fungal taxonomy [3]. For the comparisons we used a taxonomy associated with RDP database of 16S rRNA (Bacteria and Archaea) and 28S rRNA (Fungi) sequences as released on 30/09/2016 (release 11.5).

#### Greengenes (GG)

The Greengenes taxonomy [6] is dedicated to Bacteria and Archaea. Classification is based on automatic *de novo* tree construction and rank mapping from other taxonomy sources (mainly NCBI). Phylogenetic tree is constructed from 16S rRNA sequences that have been obtained from public databases and passed a quality filtering. Sequences are aligned by their characters and secondary structure and then subjected to tree construction with FastTree [20]. Inner nodes are automatically assigned taxonomic ranks from NCBI supplemented with previous version of Greengenes taxonomy and CyanoDB [21]. For the comparisons we used a taxonomy associated with the Greengenes database as released on May 2013. Although Greengenes is still included in some metagenomic analyses packages, for example QIIME [22], it has not been updated for the last three years.

#### NCBI

The NCBI taxonomy [7] contains the names of all organisms associated with submissions to the NCBI sequence databases. It is manually curated based on current systematic literature, and uses over 150 sources, for example, the *Catalog of Life* [23], the *Encyclopedia of Life* [24], *NameBank* [25] and *WikiSpecies* [26] as well as some specific databases dedicated to particular groups of organisms. It contains some duplicate names that represent different organisms. Each node has a *scientific name* and may have some *synonyms* assigned to it [7]. NCBI taxonomic classification files are updated on a daily basis; in this paper we use the version as of 05/10/2016.

#### Open tree of life taxonomy (OTT)

The Open Tree of life Taxonomy [9] aims at providing a comprehensive tree spanning as many taxa as possible. OTT is an automated synthesis of published phylogenetic trees and reference taxonomies. Phylogenetic trees have been ranked, aligned and merged together, taxonomies have been used to fill in the sparse regions and gaps left by phylogenies. Phylogenetic trees for the synthesis are obtained from TreeBASE [27], Dryad [28] and in some cases directly from contributing authors. Taxonomies are sourced from *IndexFungorum* [29], SILVA, NCBI, *Global Biodiversity Information Facility* [30], *Interim Register of Marine and Nonmarine Genera* [31] and some clade specific resources [9]. For the comparisons we used OTT taxonomy v2.10 draft 11 as generated on 10/09/2016.

## Methods

### Shared taxonomic units

First we determined how similar taxonomies are to each other by counting how many taxa they have in common at each rank. Similar comparisons have been carried out by Yilmaz et al. [4], however they confined their comparison to 16S databases, that is, SILVA, RDP and Greengenes; and only to phylum and genus levels.

**Fig. 3:**
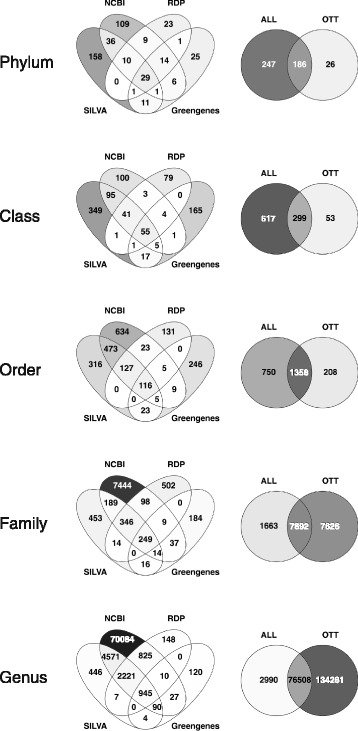
Comparison of taxonomies based on taxon names found at each rank from *phylum* to *genus*. The four taxonomies, *SILVA*, *RDP*, *Greengenes* and *NCBI*, commonly used for metagenomic analyses are compared in detail (Venn diagrams on the left) and then union of them (labeled ALL) is compared against OTT (Venn diagrams on the right). Colour intensity corresponds to the percentage of taxonomic units in the intersection. Produced with Venny 2.1 [33]

We compared the number of shared taxonomic units (by name) between the four taxonomies: SILVA, RDP, Greengenes and NCBI, at each rank from phylum to genus. We then compared the union of the four taxonomies (ALL in Fig. 3) against the OTT in the same way (See Fig. 3). To avoid differences because of alternative names or misspellings, we used the NCBI synonym dictionary to correct all names to scientific names.

### Mapping procedure

We define a procedure for mapping the nodes of one taxonomic classification onto nodes of another that is based on their hierarchical rank structure. As mentioned above, some of the taxonomies do not contain intermediate ranks, so we limit our comparisons to the seven main ranks. To get a detailed picture of how similar different pairs of taxonomies are, we perform three different mappings: *strict*, *loose* and *path comparison*, as we describe in the following.

Let *A* be source taxonomy that we are mapping into a target taxonomy *B*. Let *rank*(*a*) be a function that defines the rank of a node *a*∈*A* and *name*(*a*) be the name of *a*. We say that we can map *a*∈*A* perfectly into *B* when there is a node *b*∈*B* such that *rank*(*a*)=*rank*(*b*) and *name*(*a*)=*name*(*b*). We denote a mapping of *a* as *μ*(*a*).

Let *A* and *B* be the two taxonomies to be compared. In the following we will assume that both taxonomies contained only nodes that are assigned to one of the seven main levels. To achieve this, we preprocess each taxonomy by contracting all edges that lead to a node that is not assigned to one of the seven main ranks, thus removing all such nodes.

#### Strict mapping

**Fig. 4:**
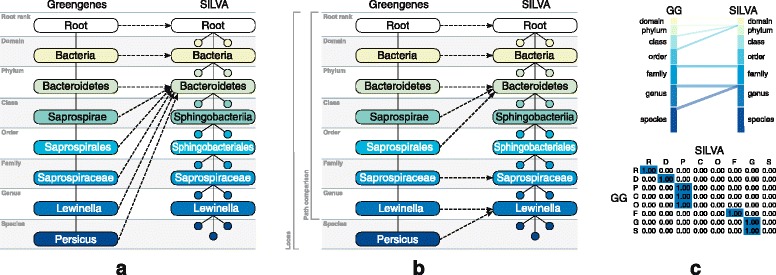
Examples of the mapping procedures (Greengenes into SILVA) on a set of nodes on the path from the Root to the species *Persicus*. **a** Strict mapping (*top–down*). From the root node we can match a path only down to the phylum level, hence all the nodes below the phylum level on the path in Greengenes are mapped to the phylum *Bacteroidetes* in SILVA. **b** Loose mapping (*bottom–up*). The node *Persicus* with species rank in Greengenes does not have a perfect match in SILVA, but its parent node *Lewinella* with genus rank has a match, therefore *Persicus* is mapped to the same node as *Lewinella*. In the path comparisons we consider only nodes that can be mapped perfectly themselves or whose descendants have perfect mappings. Here we consider the node *Lewinella* and all above, but leave out species node *Persicus*. **c** Visualization of the loose mapping from (**b**) as parallel sets and a heatmap with numeric values. Parallel sets plot show the “flow” of the mappings; the more parallel lines connecting the two bars, the better the overall mapping. Heatmap values are normalized by the row sums. A strong emphasis of the main diagonal indicates that the two taxonomies are compatible

A *strict mapping* is calculated in a pre-order traversal as follows. If some node *a* has no perfect match in *B*, then we map *a* and all of its descendants to the same node as the parent of *a*. Note that we can always map the *root* node perfectly. See Fig. 4 a for an example of a strict mapping on a set of nodes on a single path from root to species *Persicus* in Greengenes onto SILVA.

#### Loose mapping

A *loose mapping* is calculated in a pre-order traversal as follows. If some node *a*∈*A* maps perfectly to a node *b*∈*B* then we set *μ*(*a*):=*b*. Let *a* ^′^∈*A* be a node that has no perfect mapping in *B* and *a* ^″^ be an ancestral node of *a* ^′^, then we map *a* ^′^ to the same node as *a* ^″^, i.e., *μ*(*a* ^′^):=*μ*(*a* ^″^). The main difference between the two kinds of mappings is that for the loose mapping, if *a* is mapped perfectly to *b*, then we do not require that all ancestral nodes of *a* are also mapped perfectly (see Fig. 4 b).

#### Path comparison

Path comparison is a special case of the loose mapping procedure. Here we take into account only those nodes in *A* that, themselves, or whose descendants, can be mapped perfectly onto *B*. In other words, we compare the paths from the root to the nodes with the same name and the same rank (see Fig. 4 b).

### Summary dissimilarity

By applying either the strict or the loose mapping procedure, each node *a*∈*A* is mapped to some node *b*∈*B*. If the mapping is not perfect, then we will express this using a score based on the rank differences between such nodes *a* and *b*. To this end, we define the level of a rank as the distance from the root of taxonomy, that is, *level*(root)=0,*level*(domain)=1,…,*level*(genus)=6. We ignore nodes at the rank of species because the RDP and SILVA taxonomies do not contain them, whereas NCBI and OTT both have more species nodes than the others have nodes in total.

The mapping distance for node *a*∈*A* mapped to *b*∈*B* is defined as |*level*(*a*)−*level*(*b*)|, a measure of how different *A* and *B* are with respect to placement of the node *a*.

The overall dissimilarity of two taxonomies *A* and *B* is calculated as the sum of all distances normalized by the sum of maximum distances:

1 $$ Q(A,B) = \frac {\sum_{a} \left({level}(a) - {level}(\mu(a)) \right)} {\sum_{a} {level}(a)},   $$

with the sum running over all nodes *a* with *rank*(*a*)≠*species* and *μ*(*a*) denoting the node in *B* to which *a* is mapped to. Note that *level*(*a*)≥*level*(*μ*(*a*)) for all *a*∈*A*. The value of *Q*(*A,B*) lies between 0, indicating that all nodes in *A* are mapped perfectly to *B*, and 1, indicating that all nodes in *A* are mapped to the *root* of *B*, respectively. Note that the mapping dissimilarity is not symmetric, that is, in general we have *Q*(*A,B*)≠*Q*(*B,A*).

To allow a more detailed insight into the mappings, we provide summaries of the mapping results both as heat maps and as parallel sets (Fig. 4 c). We provide such visualizations for all pairs of taxonomies and all types of mapping procedures in the Additional file 1. In the “Results” section we provide an overview graph of summary dissimilarities for all pairs.

## Implementation

The mapping procedures described in this paper are implemented in a Java program called *CrossClassify*, which reads and writes taxonomic classifications in BIOM1 [32] format. The program is Open Source (AGPL license) and is available from http://ab.inf.uni-tuebingen.de/software/crossclassify/.

## Results

### Comparison by shared taxonomic units

The simple comparison reported in Fig. 3 clearly shows that there are a lot differences between the four taxonomic classifications, but there is also a lot of overlap, too. Each taxonomy at each rank has many taxa not shared with any other taxonomy – 73*%* of phyla, 70*%* of class, 63*%* of order, 90*%* of all families and 89*%* of all genera are unique to either SILVA, RDP, Greengenes or NCBI (OTT excluded). The NCBI taxonomy shares many more taxa with SILVA (60*%* in phylum, class and order ranks, and 10*%* in family and genus ranks) than it does with RDP (23*%* and 5*%*) or Greengenes (13*%* and 2*%*).

Interestingly, there are not many taxonomic units in the intersections that exclude NCBI (6*%* of phyla, 3*%* class, 1*%* order and <1*%* of families and genera), indicating that the other three taxonomies are mostly contained in the NCBI taxonomy. Comparing the unions of the four taxonomies against OTT, an immediate conclusion is that there is more variety in the union (ALL) at the phylum (54*%*) and class (64*%*) ranks, and more in OTT at the genus (63*%*) and species ranks (not shown), whereas at order and family ranks highest portion of taxonomic units is shared among the union (ALL) and OTT (59*%* and 46*%* accordingly).

### Comparison by mapping

**Fig. 5:**
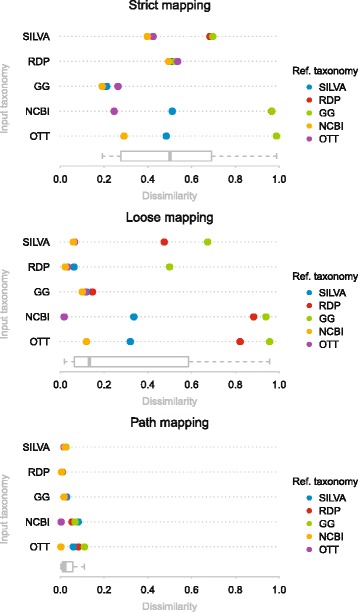
Dissimilarities between the five taxonomies based on the pairwise mappings as estimated using formula 1. Box plots under each plot show distribution of all scores for each mapping procedure

We compare the five taxonomic classifications by mapping them onto each other using the three methods defined above. In Fig. 5 we show mapping scores for all pairs of taxonomies.

The strict mapping procedure gives very poor scores for most of the pairs with median dissimilarity of 0.5. Dissimilarities lower than 0.28 (25th percentile) are observed only for the mappings of Greengenes onto other taxonomies and for the mapping of NCBI onto OTT.

The loose mapping algorithm allows one to map nodes to closer ranks when possible and this is reflected in the dissimilarity distribution (median dissimilarity 0.13) as shown in Fig. 5. Loose mapping identifies RDP and Greengenes as the most difficult to map to with average dissimilarity of 0.58 for mappings on RDP and 0.77 for Greengenes. Loose mappings onto SILVA taxonomy have an average dissimilarity of 0.21 which is much better than for RDP and Greengenes, but not as good as for the two largest taxonomies – NCBI and OTT which have average mapping dissimilarities of 0.08 and 0.06 accordingly. However when mapping NCBI and OTT onto other taxonomies we get much worse average dissimilarities of approximately 0.68 for both. In fact, the NCBI taxonomy maps much better onto OTT (dissimilarity of 0.02) than vice versa (0.12).

The mapping of the common paths produces much lower dissimilarities (median 0.02) than the loose mapping procedure, albeit not perfect. That is, there is some disagreement between the taxonomies on the paths to the same taxonomic units.

### Other applications

**Fig. 6:**
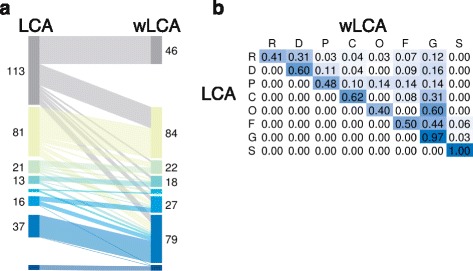
Difference between taxonomic assignment with LCA and weighted LCA. Both plots indicate more specific assignments by weighted LCA as compared to LCA. Bars in the parallel sets plot in **a** correspond to the ranks from top as follows: root, domain, phylum, class, order, family, genus and species. Columns and rows in the heatmap in **b** correspond to the same ranks: R (root), D (domain), P (phylum), C (class), O (order), F (family), G (genus) and S (species)

The scoring functions and visualization techniques introduced in this paper to compare taxonomies can be used for other purposes, too. For example, they can be used to compare the behavior of two different taxonomic binning methods on the same set of sequencing reads and (same) taxonomy. In this context, the mapping function is defined by the two taxonomic assignments of each read. In a parallel sets plot, we scale the bars so that each bar is proportional to the number of reads that are mapped to the corresponding rank. For example, in Fig. 6 we display a comparison of the naive *Lowest Common Ancestor* (LCA) algorithm and the so-called *weighted LCA* (wLCA) algorithm [1], clearly showing that one method produces more specific taxonomic assignments than the other.

## Discussion and conclusions

Comparing taxonomies by shared taxonomic units as shown in Fig. 3, we find that the number in the intersections is strongly limited by the size of the smallest taxonomy, Greengenes (see Table 1). SILVA, being the largest of the three 16S based taxonomies, shares the most taxonomic units with NCBI. We find that results of these simple comparisons are dominated by the number of nodes in each taxonomy and they tell us very little about structural (topological) compatibility of the taxonomic classifications. We address this issue by mapping taxonomies onto each other.

Our strict mapping procedure indicates how compatible the cores of taxonomic classifications are. Loose mapping on the other hand, has a less conservative nature and is closer to the comparison of shared taxonomic units. It indicates overall compatibility between taxonomies disregarding discrepancies at higher ranks that appear to be quite common; the median mapping dissimilarity for strict mapping is almost four (3.76) times as large as for the loose mapping, as shown in the box plots in Fig. 5.

The large difference between strict and loose mapping dissimilarities for NCBI to OTT (Fig. 5 and Additional file 1: Table S1) indicates that there are a few nodes with high rank that are incompatible between NCBI and OTT, but overall the two taxonomies are very similar (see Additional file 1 for more details). A much worse dissimilarity for loose mapping of OTT to NCBI (0.12) is most likely due to the fact that OTT has almost twice as many nodes as does NCBI.

Small differences (<0.05) between strict and loose mapping dissimilarities are mostly observed for the pairs of taxonomies where both dissimilarities are relatively high (>0.5). This is the case for all mappings on Greengenes. Again, this is not surprising since Greengenes is the smallest taxonomic classification and such differences indicate that it is much less diverse than other taxonomies. Dissimilarity for the loose mapping of OTT to NCBI is quite small (0.12) indicating that even though OTT is twice as large as NCBI, it is not much more diverse. This observation is also supported by differences in the number of nodes at each rank (Fig. 2, Additional file 1: Table S2) — the numbers for NCBI and OTT are very similar up to the family rank and differ significantly from genus rank only.

Path comparison dissimilarities indicate the scale of differences among paths to the taxonomic units shared pairwise by these taxonomies. Ideally all paths would be the same and their dissimilarities equal to 0. However, in this case there should also be no difference between the results of strict and loose mappings. Path comparisons show exactly how much difference there is in “shared” structure. Results (as shown in Fig. 5) show the same trend as discovered above – SILVA, RDP and Greengenes map well on NCBI and OTT but not vice versa. NCBI and OTT both are very similar with respect to path comparisons (dissimilarities are <0.01).

Because OTT is the largest of the five taxonomies and because all other taxonomies map well on it, one might consider using OTT as the reference taxonomy of choice. However, at present OTT has no sequence database associated with it, which limits its usefulness in the context of metagenomics. Therefore, we recommend using the NCBI taxonomy as a common framework when comparing analyses performed on different taxonomic classifications. While the SILVA taxonomy is widely used for 16S studies, one should consider using the NCBI taxonomy in studies that use both targeted 16S sequencing and shotgun sequencing.

## Additional file

Additional file 1 Supplementary material. A PDF file containing supporting data for the figures and detailed visualizations of pairwise mappings. (PDF 197 kb)

## Abbreviations

GG: Greengenes
LCA: Lowest common ancestor
NCBI: National Center for Biotechnology Information
OTT: Open tree of life taxonomy
RDP: Ribosomal database project
wLCA: Weighted LCA
