# Supplementary material for: SILVA, RDP, Greengenes, NCBI and OTT — how do these taxonomies compare?
Source: BMC Genomics. 2017 Mar 14;18(Suppl 2):114. doi: 10.1186/s12864-017-3501-4 (PMC5374703; doi:10.1186/s12864-017-3501-4)
Supplement: Supplementary file 1 — Supplementary material. A PDF file containing supporting data for the figures and detailed visualizations of pairwise mappings. (PDF 197 kb) [file 12864_2017_3501_MOESM1_ESM.pdf]

Supplementary material for SILVA, RDP, Greengenes, NCBI and OTT — How do these taxonomies compare?

Part A: Mapping dissimilarities

	Strict	Loose	Path	Sparklines
RDP to SILVA	0.51	0.06	0.01	
Greengenes to SILVA	0.21	0.12	0.03	
NCBI to SILVA	0.51	0.34	0.08	
OTT to SILVA	0.48	0.32	0.06	
SILVA to RDP	0.68	0.48	0.02	
Greengenes to RDP	0.21	0.15	0.02	
NCBI to RDP	0.97	0.88	0.05	
OTT to RDP	0.99	0.82	0.08	
SILVA to Greengenes	0.70	0.67	0.03	
RDP to Greengenes	0.53	0.50	0.01	
NCBI to Greengenes	0.97	0.94	0.07	
OTT to Greengenes	0.99	0.96	0.11	
SILVA to NCBI	0.40	0.06	0.02	
RDP to NCBI	0.50	0.02	0.01	
Greengenes to NCBI	0.19	0.10	0.02	
OTT to NCBI	0.29	0.12	0.00	
SILVA to OTT	0.42	0.06	0.02	
RDP to OTT	0.54	0.03	0.01	
Greengenes to OTT	0.26	0.11	0.02	
NCBI to OTT	0.25	0.02	0.00	

Table S1: Mapping dissimilarities for all pars of taxonomies. Sparklines indicate the magnitudes of strict, loose and path mapping dissimilarities for each pair of taxonomies and are provided as a quick overview of the table.

	SILVA	RDP	Greengenes	NCBI	OTT
Root	1	1	1	1	1
Domain	3	8	2	5	3
Phylum	256	87	88	214	213
Class	585	186	248	304	357
Order	1126	403	404	1392	1575
Family	1409	1227	513	8391	15584
Genus	8529	4175	1200	79573	214991

Table S2: Number of nodes at each rank in the five taxonomies.

Part B: Detailed mapping summaries

Below we provide parallel sets plots and heatmaps that give a broader overview of each mapping than a summary score as shown in the main publication.

Parallel sets

Bars in the parallel sets plots are of constant size for each rank and the actual number of nodes is indicated by the number on the side. Parallel flow lines indicate perfect mappings whereas lines going upwards show mapping to higher ranks. Ranks are displayed in the same order in all of the plots (from the top): domain, phylum, class, order, family, genus, species.

Heatmaps

Values in heatmaps are normalized by each row. Bright diagonal indicates that most of the nodes were mapped to the same rank, whereas a column popping out signifies high incompatibility below the rank associated with the column.

SILVA to RDP

Strict mapping

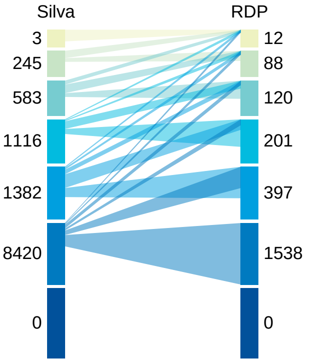

RDP

Silva	R	D	P	C	O	F	G	S
R	1.00	0.00	0.00	0.00	0.00	0.00	0.00	0.00
D	0.33	0.67	0.00	0.00	0.00	0.00	0.00	0.00
P	0.58	0.28	0.14	0.00	0.00	0.00	0.00	0.00
C	0.54	0.11	0.24	0.11	0.00	0.00	0.00	0.00
O	0.67	0.02	0.02	0.17	0.11	0.00	0.00	0.00
F	0.43	0.02	0.03	0.09	0.26	0.17	0.00	0.00
G	0.62	0.01	0.02	0.06	0.03	0.07	0.18	0.00
S	0.00	0.00	0.00	0.00	0.00	0.00	0.00	0.00

Loose mapping

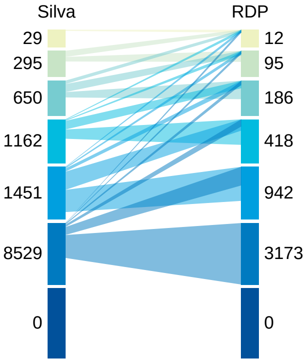

RDP

Silva	R	D	P	C	O	F	G	S
R	1.00	0.00	0.00	0.00	0.00	0.00	0.00	0.00
D	0.93	0.07	0.00	0.00	0.00	0.00	0.00	0.00
P	0.59	0.23	0.17	0.00	0.00	0.00	0.00	0.00
C	0.52	0.10	0.23	0.15	0.00	0.00	0.00	0.00
O	0.56	0.02	0.02	0.20	0.21	0.00	0.00	0.00
F	0.14	0.01	0.02	0.06	0.35	0.42	0.00	0.00
G	0.43	0.00	0.01	0.02	0.04	0.13	0.37	0.00
S	0.00	0.00	0.00	0.00	0.00	0.00	0.00	0.00

Path comparison

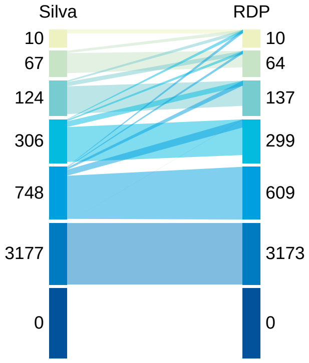

RDP

Silva	R	D	P	C	O	F	G	S
R	1.00	0.00	0.00	0.00	0.00	0.00	0.00	0.00
D	0.80	0.20	0.00	0.00	0.00	0.00	0.00	0.00
P	0.15	0.09	0.76	0.00	0.00	0.00	0.00	0.00
C	0.05	0.04	0.12	0.79	0.00	0.00	0.00	0.00
O	0.03	0.01	0.03	0.13	0.79	0.00	0.00	0.00
F	0.01	0.01	0.01	0.04	0.11	0.82	0.00	0.00
G	0.00	0.00	0.00	0.00	0.00	0.00	1.00	0.00
S	0.00	0.00	0.00	0.00	0.00	0.00	0.00	0.00

RDP to SILVA

Strict mapping

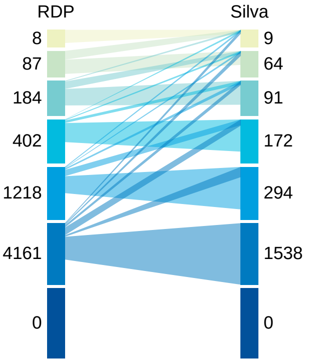

Silva

RDP	R	D	P	C	O	F	G	S
R	1.00	0.00	0.00	0.00	0.00	0.00	0.00	0.00
D	0.25	0.75	0.00	0.00	0.00	0.00	0.00	0.00
P	0.13	0.33	0.54	0.00	0.00	0.00	0.00	0.00
C	0.30	0.02	0.21	0.47	0.00	0.00	0.00	0.00
O	0.49	0.00	0.02	0.06	0.43	0.00	0.00	0.00
F	0.50	0.02	0.01	0.03	0.12	0.32	0.00	0.00
G	0.41	0.01	0.02	0.04	0.12	0.02	0.37	0.00
S	0.00	0.00	0.00	0.00	0.00	0.00	0.00	0.00

Loose mapping

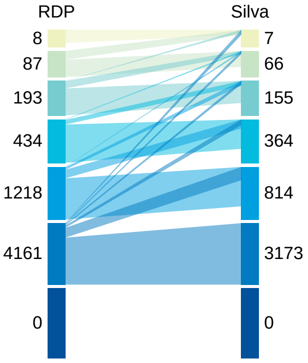

Silva

RDP	R	D	P	C	O	F	G	S
R	1.00	0.00	0.00	0.00	0.00	0.00	0.00	0.00
D	0.25	0.75	0.00	0.00	0.00	0.00	0.00	0.00
P	0.01	0.33	0.66	0.00	0.00	0.00	0.00	0.00
C	0.02	0.01	0.21	0.77	0.00	0.00	0.00	0.00
O	0.01	0.00	0.01	0.10	0.87	0.00	0.00	0.00
F	0.00	0.00	0.01	0.05	0.16	0.78	0.00	0.00
G	0.00	0.01	0.01	0.02	0.04	0.16	0.76	0.00
S	0.00	0.00	0.00	0.00	0.00	0.00	0.00	0.00

Path comparison

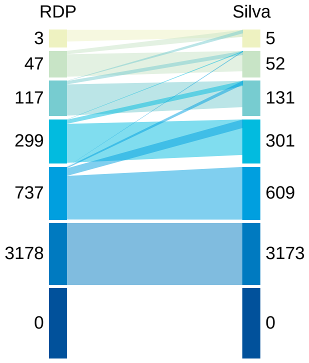

Silva

RDP	R	D	P	C	O	F	G	S
R	1.00	0.00	0.00	0.00	0.00	0.00	0.00	0.00
D	0.33	0.67	0.00	0.00	0.00	0.00	0.00	0.00
P	0.02	0.13	0.85	0.00	0.00	0.00	0.00	0.00
C	0.01	0.01	0.09	0.89	0.00	0.00	0.00	0.00
O	0.01	0.00	0.01	0.09	0.89	0.00	0.00	0.00
F	0.00	0.00	0.00	0.02	0.14	0.83	0.00	0.00
G	0.00	0.00	0.00	0.00	0.00	0.00	1.00	0.00
S	0.00	0.00	0.00	0.00	0.00	0.00	0.00	0.00

SILVA to Greengenes

Strict mapping

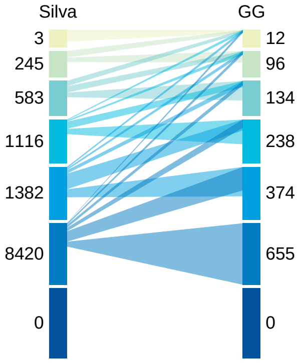

Silva (rows) / GG (columns)

	R	D	P	C	O	F	G	S
R	1.00	0.00	0.00	0.00	0.00	0.00	0.00	0.00
D	0.33	0.67	0.00	0.00	0.00	0.00	0.00	0.00
P	0.58	0.25	0.17	0.00	0.00	0.00	0.00	0.00
C	0.54	0.15	0.18	0.13	0.00	0.00	0.00	0.00
O	0.67	0.02	0.03	0.16	0.12	0.00	0.00	0.00
F	0.43	0.03	0.03	0.07	0.30	0.15	0.00	0.00
G	0.62	0.01	0.03	0.04	0.06	0.17	0.08	0.00
S	0.00	0.00	0.00	0.00	0.00	0.00	0.00	0.00

Loose mapping

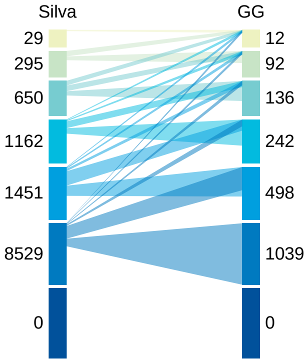

Silva (rows) / GG (columns)

	R	D	P	C	O	F	G	S
R	1.00	0.00	0.00	0.00	0.00	0.00	0.00	0.00
D	0.93	0.07	0.00	0.00	0.00	0.00	0.00	0.00
P	0.65	0.21	0.14	0.00	0.00	0.00	0.00	0.00
C	0.58	0.14	0.16	0.12	0.00	0.00	0.00	0.00
O	0.68	0.02	0.03	0.15	0.12	0.00	0.00	0.00
F	0.45	0.01	0.02	0.04	0.27	0.19	0.00	0.00
G	0.63	0.00	0.01	0.01	0.02	0.21	0.12	0.00
S	0.00	0.00	0.00	0.00	0.00	0.00	0.00	0.00

Path comparison

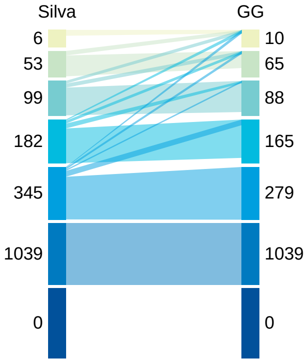

Silva (rows) / GG (columns)

	R	D	P	C	O	F	G	S
R	1.00	0.00	0.00	0.00	0.00	0.00	0.00	0.00
D	0.67	0.33	0.00	0.00	0.00	0.00	0.00	0.00
P	0.04	0.17	0.79	0.00	0.00	0.00	0.00	0.00
C	0.02	0.08	0.11	0.79	0.00	0.00	0.00	0.00
O	0.01	0.03	0.07	0.11	0.79	0.00	0.00	0.00
F	0.01	0.01	0.04	0.03	0.11	0.81	0.00	0.00
G	0.00	0.00	0.00	0.00	0.00	0.00	1.00	0.00
S	0.00	0.00	0.00	0.00	0.00	0.00	0.00	0.00

Greengenes to SILVA

Strict mapping

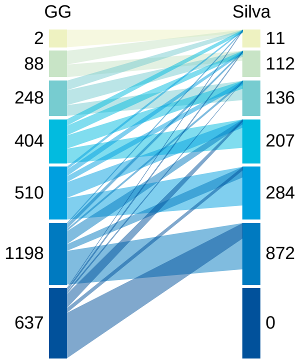

GG (rows) / Silva (columns)

	R	D	P	C	O	F	G	S
R	1.00	0.00	0.00	0.00	0.00	0.00	0.00	0.00
D	0.00	1.00	0.00	0.00	0.00	0.00	0.00	0.00
P	0.00	0.52	0.48	0.00	0.00	0.00	0.00	0.00
C	0.00	0.27	0.42	0.30	0.00	0.00	0.00	0.00
O	0.00	0.15	0.22	0.30	0.33	0.00	0.00	0.00
F	0.00	0.06	0.12	0.11	0.30	0.41	0.00	0.00
G	0.00	0.02	0.08	0.04	0.21	0.11	0.55	0.00
S	0.00	0.02	0.04	0.03	0.20	0.07	0.65	0.00

Loose mapping

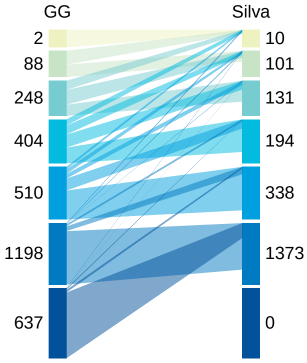

GG (rows) / Silva (columns)

	R	D	P	C	O	F	G	S
R	1.00	0.00	0.00	0.00	0.00	0.00	0.00	0.00
D	0.00	1.00	0.00	0.00	0.00	0.00	0.00	0.00
P	0.00	0.52	0.48	0.00	0.00	0.00	0.00	0.00
C	0.00	0.27	0.42	0.31	0.00	0.00	0.00	0.00
O	0.00	0.14	0.21	0.30	0.36	0.00	0.00	0.00
F	0.00	0.05	0.09	0.09	0.23	0.55	0.00	0.00
G	0.00	0.01	0.01	0.00	0.03	0.08	0.87	0.00
S	0.00	0.00	0.01	0.00	0.02	0.04	0.94	0.00

Path comparison

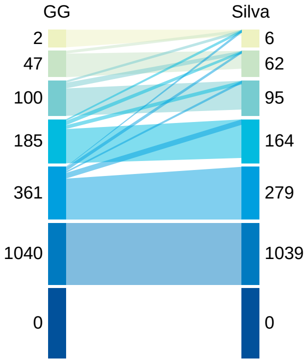

GG (rows) / Silva (columns)

	R	D	P	C	O	F	G	S
R	1.00	0.00	0.00	0.00	0.00	0.00	0.00	0.00
D	0.00	1.00	0.00	0.00	0.00	0.00	0.00	0.00
P	0.00	0.11	0.89	0.00	0.00	0.00	0.00	0.00
C	0.00	0.06	0.16	0.78	0.00	0.00	0.00	0.00
O	0.00	0.04	0.10	0.09	0.78	0.00	0.00	0.00
F	0.00	0.01	0.07	0.03	0.11	0.78	0.00	0.00
G	0.00	0.00	0.00	0.00	0.00	0.00	1.00	0.00
S	0.00	0.00	0.00	0.00	0.00	0.00	0.00	0.00

SILVA to NCBI

Strict mapping

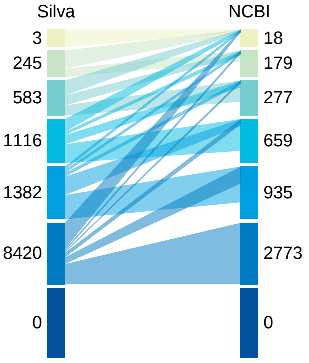

Silva \ NCBI	R	D	P	C	O	F	G	S
R	1.00	0.00	0.00	0.00	0.00	0.00	0.00	0.00
D	0.00	1.00	0.00	0.00	0.00	0.00	0.00	0.00
P	0.00	0.69	0.31	0.00	0.00	0.00	0.00	0.00
C	0.00	0.43	0.28	0.29	0.00	0.00	0.00	0.00
O	0.00	0.29	0.07	0.21	0.43	0.00	0.00	0.00
F	0.00	0.06	0.06	0.07	0.35	0.46	0.00	0.00
G	0.00	0.43	0.04	0.02	0.07	0.11	0.33	0.00
S	0.00	0.00	0.00	0.00	0.00	0.00	0.00	0.00

Loose mapping

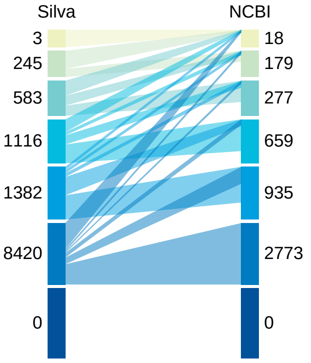

Silva \ NCBI	R	D	P	C	O	F	G	S
R	1.00	0.00	0.00	0.00	0.00	0.00	0.00	0.00
D	0.00	1.00	0.00	0.00	0.00	0.00	0.00	0.00
P	0.00	0.69	0.31	0.00	0.00	0.00	0.00	0.00
C	0.00	0.43	0.28	0.29	0.00	0.00	0.00	0.00
O	0.00	0.29	0.07	0.21	0.43	0.00	0.00	0.00
F	0.00	0.06	0.06	0.07	0.35	0.46	0.00	0.00
G	0.00	0.43	0.04	0.02	0.07	0.11	0.33	0.00
S	0.00	0.00	0.00	0.00	0.00	0.00	0.00	0.00

Path comparison

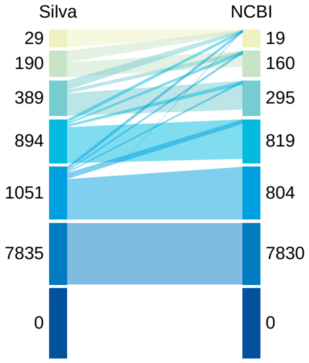

Silva \ NCBI	R	D	P	C	O	F	G	S
R	1.00	0.00	0.00	0.00	0.00	0.00	0.00	0.00
D	0.00	1.00	0.00	0.00	0.00	0.00	0.00	0.00
P	0.00	0.45	0.55	0.00	0.00	0.00	0.00	0.00
C	0.00	0.27	0.09	0.64	0.00	0.00	0.00	0.00
O	0.00	0.09	0.04	0.05	0.82	0.00	0.00	0.00
F	0.00	0.07	0.03	0.02	0.11	0.76	0.00	0.00
G	0.00	0.00	0.00	0.00	0.00	0.00	1.00	0.00
S	0.00	0.00	0.00	0.00	0.00	0.00	0.00	0.00

NCBI to SILVA

Strict mapping

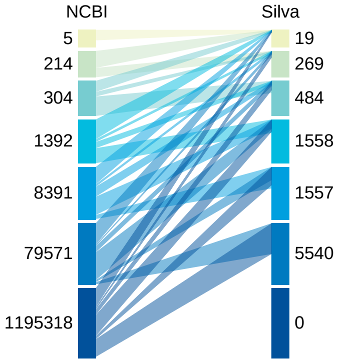

NCBI \ Silva	R	D	P	C	O	F	G	S
R	1.00	0.00	0.00	0.00	0.00	0.00	0.00	0.00
D	0.40	0.60	0.00	0.00	0.00	0.00	0.00	0.00
P	0.00	0.64	0.36	0.00	0.00	0.00	0.00	0.00
C	0.00	0.32	0.13	0.55	0.00	0.00	0.00	0.00
O	0.01	0.45	0.06	0.14	0.34	0.00	0.00	0.00
F	0.01	0.33	0.05	0.19	0.35	0.08	0.00	0.00
G	0.01	0.35	0.02	0.10	0.45	0.03	0.03	0.00
S	0.02	0.27	0.03	0.08	0.30	0.04	0.25	0.00

Loose mapping

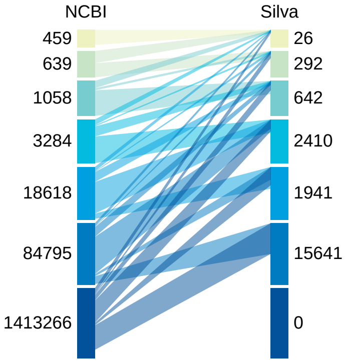

NCBI \ Silva	R	D	P	C	O	F	G	S
R	1.00	0.00	0.00	0.00	0.00	0.00	0.00	0.00
D	0.14	0.86	0.00	0.00	0.00	0.00	0.00	0.00
P	0.00	0.46	0.54	0.00	0.00	0.00	0.00	0.00
C	0.00	0.21	0.06	0.73	0.00	0.00	0.00	0.00
O	0.00	0.13	0.02	0.23	0.61	0.00	0.00	0.00
F	0.02	0.08	0.01	0.19	0.60	0.10	0.00	0.00
G	0.01	0.06	0.01	0.16	0.60	0.04	0.13	0.00
S	0.12	0.08	0.02	0.08	0.32	0.04	0.35	0.00

Path comparison

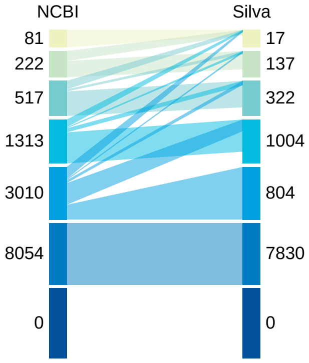

NCBI \ Silva	R	D	P	C	O	F	G	S
R	1.00	0.00	0.00	0.00	0.00	0.00	0.00	0.00
D	0.00	1.00	0.00	0.00	0.00	0.00	0.00	0.00
P	0.00	0.38	0.62	0.00	0.00	0.00	0.00	0.00
C	0.00	0.24	0.06	0.70	0.00	0.00	0.00	0.00
O	0.00	0.18	0.02	0.09	0.71	0.00	0.00	0.00
F	0.00	0.23	0.02	0.05	0.41	0.28	0.00	0.00
G	0.00	0.00	0.00	0.00	0.00	0.00	1.00	0.00
S	0.00	0.00	0.00	0.00	0.00	0.00	0.00	0.00

SILVA to OTT

Strict mapping

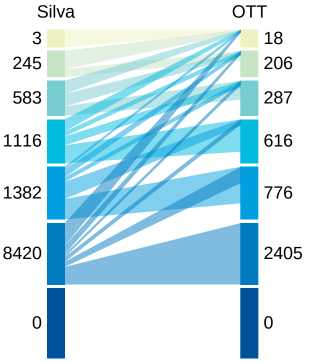

OTT

Silva	R	D	P	C	O	F	G	S
R	1.00	0.00	0.00	0.00	0.00	0.00	0.00	0.00
D	0.00	1.00	0.00	0.00	0.00	0.00	0.00	0.00
P	0.00	0.70	0.30	0.00	0.00	0.00	0.00	0.00
C	0.00	0.37	0.36	0.27	0.00	0.00	0.00	0.00
O	0.00	0.28	0.11	0.20	0.41	0.00	0.00	0.00
F	0.00	0.05	0.15	0.09	0.33	0.39	0.00	0.00
G	0.00	0.37	0.14	0.05	0.08	0.08	0.29	0.00
S	0.00	0.00	0.00	0.00	0.00	0.00	0.00	0.00

Loose mapping

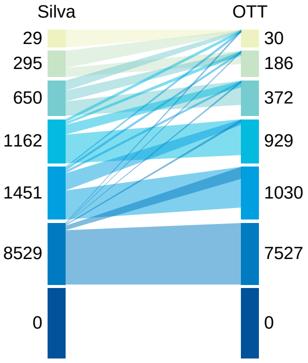

OTT

Silva	R	D	P	C	O	F	G	S
R	1.00	0.00	0.00	0.00	0.00	0.00	0.00	0.00
D	0.00	1.00	0.00	0.00	0.00	0.00	0.00	0.00
P	0.00	0.66	0.34	0.00	0.00	0.00	0.00	0.00
C	0.00	0.29	0.30	0.41	0.00	0.00	0.00	0.00
O	0.00	0.07	0.05	0.22	0.65	0.00	0.00	0.00
F	0.00	0.03	0.05	0.05	0.33	0.55	0.00	0.00
G	0.00	0.01	0.01	0.01	0.02	0.07	0.88	0.00
S	0.00	0.00	0.00	0.00	0.00	0.00	0.00	0.00

Path comparison

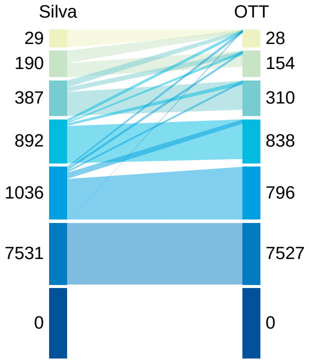

OTT

Silva	R	D	P	C	O	F	G	S
R	1.00	0.00	0.00	0.00	0.00	0.00	0.00	0.00
D	0.00	1.00	0.00	0.00	0.00	0.00	0.00	0.00
P	0.00	0.47	0.53	0.00	0.00	0.00	0.00	0.00
C	0.00	0.20	0.13	0.68	0.00	0.00	0.00	0.00
O	0.00	0.07	0.03	0.05	0.85	0.00	0.00	0.00
F	0.00	0.03	0.05	0.03	0.12	0.77	0.00	0.00
G	0.00	0.00	0.00	0.00	0.00	0.00	1.00	0.00
S	0.00	0.00	0.00	0.00	0.00	0.00	0.00	0.00

OTT to SILVA

Strict mapping

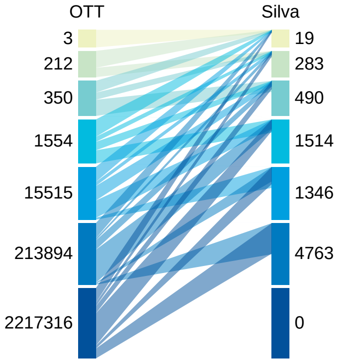

Silva

OTT	R	D	P	C	O	F	G	S
R	1.00	0.00	0.00	0.00	0.00	0.00	0.00	0.00
D	0.00	1.00	0.00	0.00	0.00	0.00	0.00	0.00
P	0.00	0.64	0.35	0.00	0.00	0.00	0.00	0.00
C	0.00	0.34	0.20	0.46	0.00	0.00	0.00	0.00
O	0.00	0.40	0.11	0.19	0.30	0.00	0.00	0.00
F	0.00	0.29	0.08	0.28	0.33	0.03	0.00	0.00
G	0.00	0.25	0.04	0.15	0.54	0.02	0.01	0.00
S	0.00	0.21	0.05	0.10	0.46	0.04	0.14	0.00

Loose mapping

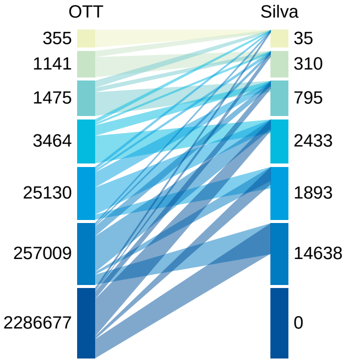

Silva

OTT	R	D	P	C	O	F	G	S
R	1.00	0.00	0.00	0.00	0.00	0.00	0.00	0.00
D	0.00	1.00	0.00	0.00	0.00	0.00	0.00	0.00
P	0.00	0.24	0.76	0.00	0.00	0.00	0.00	0.00
C	0.00	0.20	0.13	0.67	0.00	0.00	0.00	0.00
O	0.00	0.09	0.05	0.24	0.63	0.00	0.00	0.00
F	0.00	0.06	0.04	0.29	0.51	0.09	0.00	0.00
G	0.00	0.03	0.02	0.16	0.58	0.04	0.16	0.00
S	0.00	0.04	0.01	0.11	0.53	0.04	0.27	0.00

Path comparison

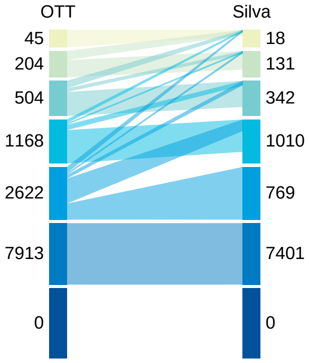

Silva

OTT	R	D	P	C	O	F	G	S
R	1.00	0.00	0.00	0.00	0.00	0.00	0.00	0.00
D	0.00	1.00	0.00	0.00	0.00	0.00	0.00	0.00
P	0.00	0.35	0.65	0.00	0.00	0.00	0.00	0.00
C	0.00	0.21	0.11	0.68	0.00	0.00	0.00	0.00
O	0.00	0.08	0.04	0.12	0.76	0.00	0.00	0.00
F	0.00	0.12	0.03	0.07	0.47	0.30	0.00	0.00
G	0.00	0.00	0.00	0.00	0.00	0.00	1.00	0.00
S	0.00	0.00	0.00	0.00	0.00	0.00	0.00	0.00

RDP to Greengenes

Strict mapping

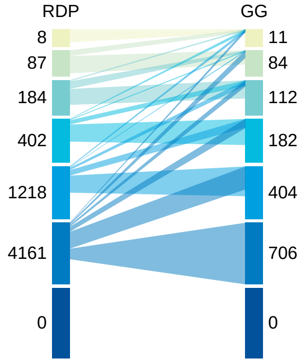

GG

RDP	R	D	P	C	O	F	G	S
R	1.00	0.00	0.00	0.00	0.00	0.00	0.00	0.00
D	0.25	0.75	0.00	0.00	0.00	0.00	0.00	0.00
P	0.13	0.22	0.66	0.00	0.00	0.00	0.00	0.00
C	0.30	0.03	0.22	0.45	0.00	0.00	0.00	0.00
O	0.49	0.01	0.02	0.09	0.39	0.00	0.00	0.00
F	0.50	0.02	0.01	0.04	0.10	0.32	0.00	0.00
G	0.41	0.01	0.02	0.03	0.08	0.27	0.17	0.00
S	0.00	0.00	0.00	0.00	0.00	0.00	0.00	0.00

Loose mapping

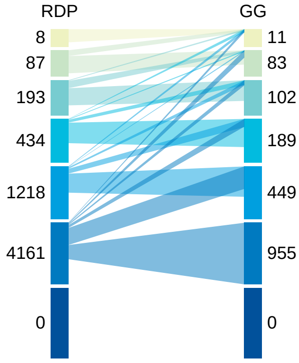

GG

RDP	R	D	P	C	O	F	G	S
R	1.00	0.00	0.00	0.00	0.00	0.00	0.00	0.00
D	0.25	0.75	0.00	0.00	0.00	0.00	0.00	0.00
P	0.13	0.22	0.66	0.00	0.00	0.00	0.00	0.00
C	0.28	0.02	0.21	0.49	0.00	0.00	0.00	0.00
O	0.45	0.00	0.02	0.06	0.47	0.00	0.00	0.00
F	0.50	0.01	0.01	0.03	0.09	0.36	0.00	0.00
G	0.41	0.01	0.02	0.02	0.05	0.28	0.23	0.00
S	0.00	0.00	0.00	0.00	0.00	0.00	0.00	0.00

Path comparison

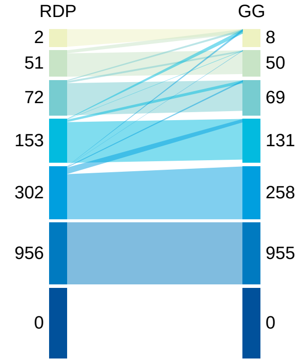

GG

RDP	R	D	P	C	O	F	G	S
R	1.00	0.00	0.00	0.00	0.00	0.00	0.00	0.00
D	0.00	1.00	0.00	0.00	0.00	0.00	0.00	0.00
P	0.00	0.12	0.88	0.00	0.00	0.00	0.00	0.00
C	0.00	0.03	0.06	0.92	0.00	0.00	0.00	0.00
O	0.00	0.01	0.01	0.05	0.93	0.00	0.00	0.00
F	0.00	0.00	0.00	0.02	0.12	0.85	0.00	0.00
G	0.00	0.00	0.00	0.00	0.00	0.00	1.00	0.00
S	0.00	0.00	0.00	0.00	0.00	0.00	0.00	0.00

Greengenes to RDP

Strict mapping

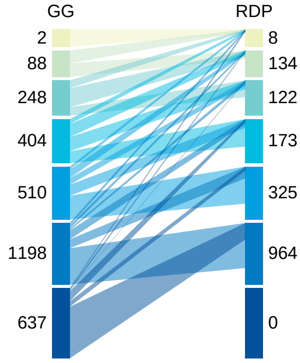

RDP

GG	R	D	P	C	O	F	G	S
R	1.00	0.00	0.00	0.00	0.00	0.00	0.00	0.00
D	0.00	1.00	0.00	0.00	0.00	0.00	0.00	0.00
P	0.00	0.49	0.51	0.00	0.00	0.00	0.00	0.00
C	0.00	0.22	0.54	0.23	0.00	0.00	0.00	0.00
O	0.00	0.11	0.30	0.32	0.27	0.00	0.00	0.00
F	0.00	0.06	0.16	0.12	0.21	0.45	0.00	0.00
G	0.00	0.04	0.05	0.03	0.14	0.14	0.59	0.00
S	0.00	0.02	0.03	0.00	0.14	0.08	0.73	0.00

Loose mapping

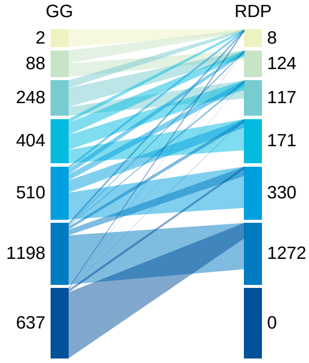

RDP

GG	R	D	P	C	O	F	G	S
R	1.00	0.00	0.00	0.00	0.00	0.00	0.00	0.00
D	0.00	1.00	0.00	0.00	0.00	0.00	0.00	0.00
P	0.00	0.49	0.51	0.00	0.00	0.00	0.00	0.00
C	0.00	0.22	0.54	0.24	0.00	0.00	0.00	0.00
O	0.00	0.09	0.27	0.34	0.30	0.00	0.00	0.00
F	0.00	0.05	0.10	0.12	0.23	0.51	0.00	0.00
G	0.00	0.04	0.01	0.01	0.05	0.09	0.80	0.00
S	0.00	0.02	0.00	0.00	0.00	0.04	0.94	0.00

Path comparison

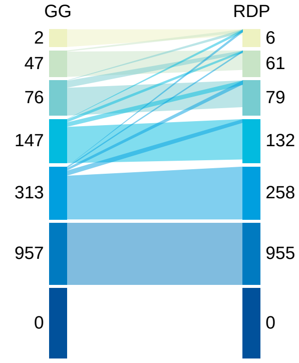

RDP

GG	R	D	P	C	O	F	G	S
R	1.00	0.00	0.00	0.00	0.00	0.00	0.00	0.00
D	0.00	1.00	0.00	0.00	0.00	0.00	0.00	0.00
P	0.00	0.04	0.96	0.00	0.00	0.00	0.00	0.00
C	0.00	0.01	0.20	0.79	0.00	0.00	0.00	0.00
O	0.00	0.01	0.07	0.10	0.82	0.00	0.00	0.00
F	0.00	0.00	0.03	0.05	0.10	0.83	0.00	0.00
G	0.00	0.00	0.00	0.00	0.00	0.00	1.00	0.00
S	0.00	0.00	0.00	0.00	0.00	0.00	0.00	0.00

RDP to NCBI

Strict mapping

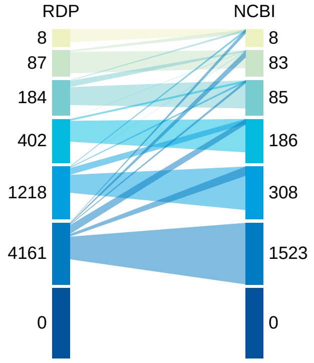

RDP \ NCBI	R	D	P	C	O	F	G	S
R	1.00	0.00	0.00	0.00	0.00	0.00	0.00	0.00
D	0.25	0.75	0.00	0.00	0.00	0.00	0.00	0.00
P	0.13	0.06	0.82	0.00	0.00	0.00	0.00	0.00
C	0.30	0.02	0.18	0.51	0.00	0.00	0.00	0.00
O	0.49	0.00	0.00	0.04	0.47	0.00	0.00	0.00
F	0.50	0.01	0.00	0.02	0.13	0.34	0.00	0.00
G	0.41	0.01	0.02	0.02	0.14	0.04	0.37	0.00
S	0.00	0.00	0.00	0.00	0.00	0.00	0.00	0.00

Loose mapping

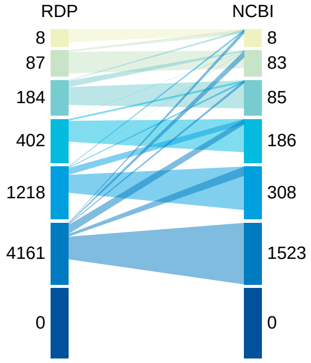

RDP \ NCBI	R	D	P	C	O	F	G	S
R	1.00	0.00	0.00	0.00	0.00	0.00	0.00	0.00
D	0.25	0.75	0.00	0.00	0.00	0.00	0.00	0.00
P	0.13	0.06	0.82	0.00	0.00	0.00	0.00	0.00
C	0.30	0.02	0.18	0.51	0.00	0.00	0.00	0.00
O	0.49	0.00	0.00	0.04	0.47	0.00	0.00	0.00
F	0.50	0.01	0.00	0.02	0.13	0.34	0.00	0.00
G	0.41	0.01	0.02	0.02	0.14	0.04	0.37	0.00
S	0.00	0.00	0.00	0.00	0.00	0.00	0.00	0.00

Path comparison

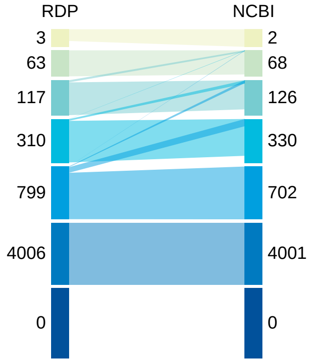

RDP \ NCBI	R	D	P	C	O	F	G	S
R	1.00	0.00	0.00	0.00	0.00	0.00	0.00	0.00
D	0.33	0.67	0.00	0.00	0.00	0.00	0.00	0.00
P	0.02	0.00	0.98	0.00	0.00	0.00	0.00	0.00
C	0.01	0.00	0.06	0.93	0.00	0.00	0.00	0.00
O	0.01	0.00	0.00	0.04	0.95	0.00	0.00	0.00
F	0.00	0.00	0.00	0.01	0.11	0.88	0.00	0.00
G	0.00	0.00	0.00	0.00	0.00	0.00	1.00	0.00
S	0.00	0.00	0.00	0.00	0.00	0.00	0.00	0.00

NCBI to RDP

Strict mapping

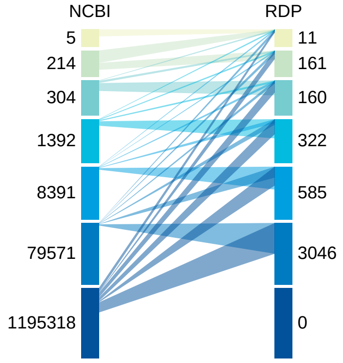

NCBI \ RDP	R	D	P	C	O	F	G	S
R	1.00	0.00	0.00	0.00	0.00	0.00	0.00	0.00
D	0.60	0.40	0.00	0.00	0.00	0.00	0.00	0.00
P	0.27	0.47	0.26	0.00	0.00	0.00	0.00	0.00
C	0.70	0.01	0.06	0.22	0.00	0.00	0.00	0.00
O	0.85	0.01	0.02	0.02	0.10	0.00	0.00	0.00
F	0.94	0.01	0.00	0.01	0.01	0.03	0.00	0.00
G	0.96	0.00	0.00	0.01	0.01	0.01	0.02	0.00
S	0.65	0.06	0.01	0.06	0.05	0.02	0.14	0.00

Loose mapping

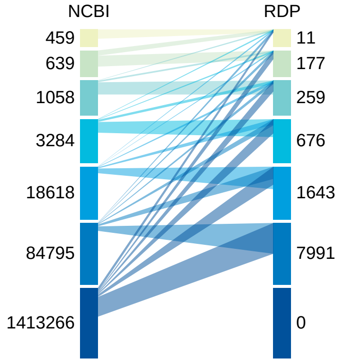

NCBI \ RDP	R	D	P	C	O	F	G	S
R	1.00	0.00	0.00	0.00	0.00	0.00	0.00	0.00
D	0.47	0.53	0.00	0.00	0.00	0.00	0.00	0.00
P	0.39	0.20	0.41	0.00	0.00	0.00	0.00	0.00
C	0.60	0.01	0.04	0.35	0.00	0.00	0.00	0.00
O	0.69	0.01	0.01	0.05	0.24	0.00	0.00	0.00
F	0.88	0.00	0.00	0.01	0.02	0.09	0.00	0.00
G	0.87	0.00	0.00	0.01	0.01	0.03	0.07	0.00
S	0.59	0.06	0.01	0.01	0.01	0.03	0.28	0.00

Path comparison

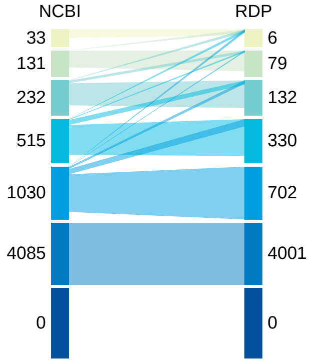

NCBI \ RDP	R	D	P	C	O	F	G	S
R	1.00	0.00	0.00	0.00	0.00	0.00	0.00	0.00
D	0.52	0.48	0.00	0.00	0.00	0.00	0.00	0.00
P	0.36	0.01	0.63	0.00	0.00	0.00	0.00	0.00
C	0.29	0.01	0.07	0.63	0.00	0.00	0.00	0.00
O	0.19	0.00	0.02	0.11	0.68	0.00	0.00	0.00
F	0.14	0.00	0.00	0.04	0.10	0.71	0.00	0.00
G	0.00	0.00	0.00	0.00	0.00	0.00	1.00	0.00
S	0.00	0.00	0.00	0.00	0.00	0.00	0.00	0.00

RDP to OTT

Strict mapping

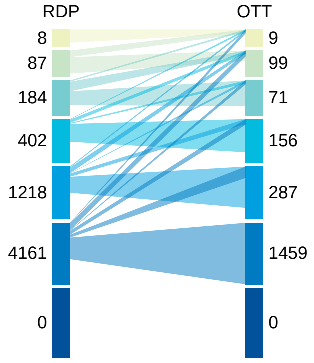

OTT

RDP	R	D	P	C	O	F	G	S
R	1.00	0.00	0.00	0.00	0.00	0.00	0.00	0.00
D	0.25	0.75	0.00	0.00	0.00	0.00	0.00	0.00
P	0.13	0.25	0.62	0.00	0.00	0.00	0.00	0.00
C	0.30	0.04	0.27	0.39	0.00	0.00	0.00	0.00
O	0.49	0.01	0.08	0.02	0.41	0.00	0.00	0.00
F	0.50	0.02	0.11	0.00	0.06	0.30	0.00	0.00
G	0.41	0.01	0.11	0.01	0.06	0.05	0.35	0.00
S	0.00	0.00	0.00	0.00	0.00	0.00	0.00	0.00

Loose mapping

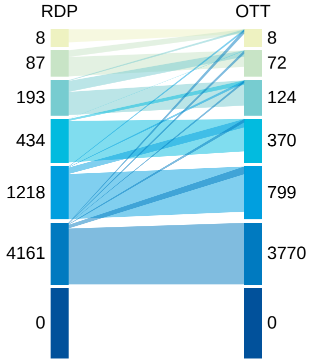

OTT

RDP	R	D	P	C	O	F	G	S
R	1.00	0.00	0.00	0.00	0.00	0.00	0.00	0.00
D	0.25	0.75	0.00	0.00	0.00	0.00	0.00	0.00
P	0.01	0.25	0.74	0.00	0.00	0.00	0.00	0.00
C	0.01	0.02	0.31	0.66	0.00	0.00	0.00	0.00
O	0.01	0.00	0.00	0.04	0.94	0.00	0.00	0.00
F	0.00	0.01	0.00	0.01	0.12	0.85	0.00	0.00
G	0.00	0.01	0.01	0.00	0.01	0.06	0.91	0.00
S	0.00	0.00	0.00	0.00	0.00	0.00	0.00	0.00

Path comparison

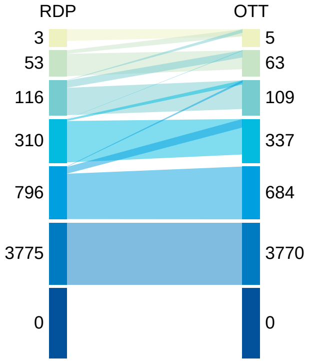

OTT

RDP	R	D	P	C	O	F	G	S
R	1.00	0.00	0.00	0.00	0.00	0.00	0.00	0.00
D	0.33	0.67	0.00	0.00	0.00	0.00	0.00	0.00
P	0.02	0.13	0.85	0.00	0.00	0.00	0.00	0.00
C	0.01	0.01	0.21	0.78	0.00	0.00	0.00	0.00
O	0.01	0.00	0.00	0.04	0.94	0.00	0.00	0.00
F	0.00	0.00	0.00	0.01	0.13	0.86	0.00	0.00
G	0.00	0.00	0.00	0.00	0.00	0.00	1.00	0.00
S	0.00	0.00	0.00	0.00	0.00	0.00	0.00	0.00

OTT to RDP

Strict mapping

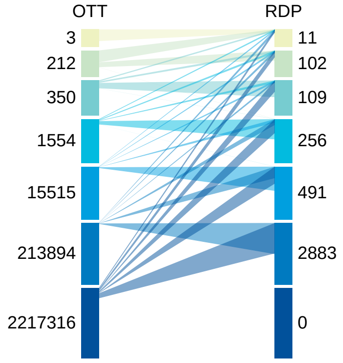

RDP

OTT	R	D	P	C	O	F	G	S
R	1.00	0.00	0.00	0.00	0.00	0.00	0.00	0.00
D	0.33	0.67	0.00	0.00	0.00	0.00	0.00	0.00
P	0.36	0.45	0.18	0.00	0.00	0.00	0.00	0.00
C	0.77	0.03	0.04	0.15	0.00	0.00	0.00	0.00
O	0.88	0.02	0.02	0.01	0.08	0.00	0.00	0.00
F	0.97	0.00	0.00	0.00	0.00	0.01	0.00	0.00
G	0.99	0.00	0.00	0.00	0.00	0.00	0.01	0.00
S	0.85	0.02	0.03	0.00	0.02	0.01	0.07	0.00

Loose mapping

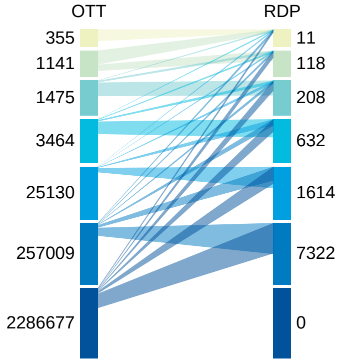

RDP

OTT	R	D	P	C	O	F	G	S
R	1.00	0.00	0.00	0.00	0.00	0.00	0.00	0.00
D	0.33	0.67	0.00	0.00	0.00	0.00	0.00	0.00
P	0.22	0.54	0.24	0.00	0.00	0.00	0.00	0.00
C	0.55	0.01	0.05	0.39	0.00	0.00	0.00	0.00
O	0.66	0.01	0.02	0.03	0.29	0.00	0.00	0.00
F	0.89	0.00	0.00	0.01	0.01	0.08	0.00	0.00
G	0.79	0.01	0.01	0.01	0.01	0.04	0.13	0.00
S	0.71	0.02	0.01	0.01	0.01	0.04	0.21	0.00

Path comparison

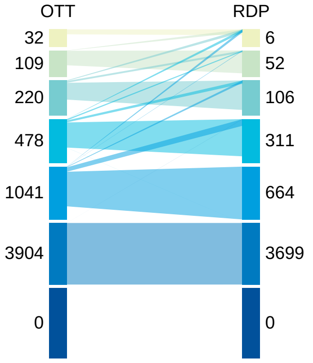

RDP

OTT	R	D	P	C	O	F	G	S
R	1.00	0.00	0.00	0.00	0.00	0.00	0.00	0.00
D	0.72	0.28	0.00	0.00	0.00	0.00	0.00	0.00
P	0.44	0.01	0.55	0.00	0.00	0.00	0.00	0.00
C	0.45	0.02	0.05	0.48	0.00	0.00	0.00	0.00
O	0.35	0.00	0.03	0.04	0.57	0.00	0.00	0.00
F	0.25	0.00	0.00	0.01	0.08	0.65	0.00	0.00
G	0.00	0.00	0.00	0.00	0.00	0.00	1.00	0.00
S	0.00	0.00	0.00	0.00	0.00	0.00	0.00	0.00

Greengenes to NCBI

Strict mapping

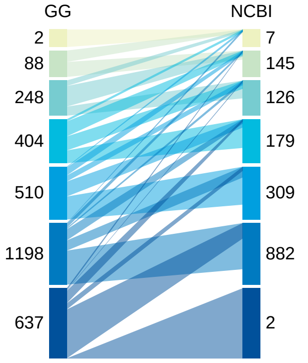

GG \ NCBI	R	D	P	C	O	F	G	S
R	1.00	0.00	0.00	0.00	0.00	0.00	0.00	0.00
D	0.00	1.00	0.00	0.00	0.00	0.00	0.00	0.00
P	0.00	0.43	0.57	0.00	0.00	0.00	0.00	0.00
C	0.00	0.16	0.58	0.26	0.00	0.00	0.00	0.00
O	0.00	0.05	0.34	0.32	0.29	0.00	0.00	0.00
F	0.00	0.01	0.15	0.11	0.28	0.44	0.00	0.00
G	0.00	0.01	0.07	0.02	0.18	0.17	0.55	0.00
S	0.00	0.00	0.03	0.01	0.18	0.09	0.69	0.00

Loose mapping

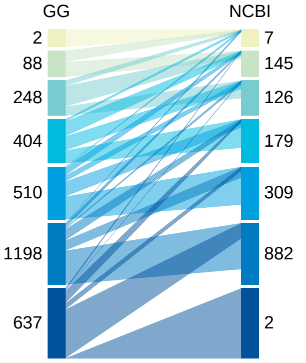

GG \ NCBI	R	D	P	C	O	F	G	S
R	1.00	0.00	0.00	0.00	0.00	0.00	0.00	0.00
D	0.00	1.00	0.00	0.00	0.00	0.00	0.00	0.00
P	0.00	0.43	0.57	0.00	0.00	0.00	0.00	0.00
C	0.00	0.16	0.58	0.26	0.00	0.00	0.00	0.00
O	0.00	0.05	0.34	0.32	0.29	0.00	0.00	0.00
F	0.00	0.01	0.15	0.11	0.28	0.44	0.00	0.00
G	0.00	0.01	0.07	0.02	0.18	0.17	0.55	0.00
S	0.00	0.00	0.03	0.01	0.18	0.09	0.69	0.00

Path comparison

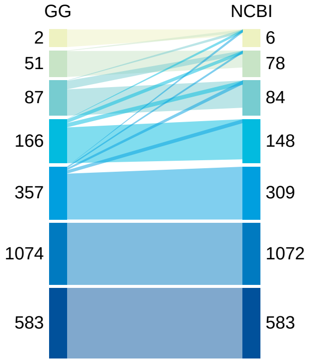

GG \ NCBI	R	D	P	C	O	F	G	S
R	1.00	0.00	0.00	0.00	0.00	0.00	0.00	0.00
D	0.00	1.00	0.00	0.00	0.00	0.00	0.00	0.00
P	0.00	0.02	0.98	0.00	0.00	0.00	0.00	0.00
C	0.00	0.01	0.24	0.75	0.00	0.00	0.00	0.00
O	0.00	0.01	0.08	0.10	0.81	0.00	0.00	0.00
F	0.00	0.00	0.03	0.04	0.06	0.87	0.00	0.00
G	0.00	0.00	0.00	0.00	0.00	0.00	1.00	0.00
S	0.00	0.00	0.00	0.00	0.00	0.00	0.00	1.00

NCBI to Greengenes

Strict mapping

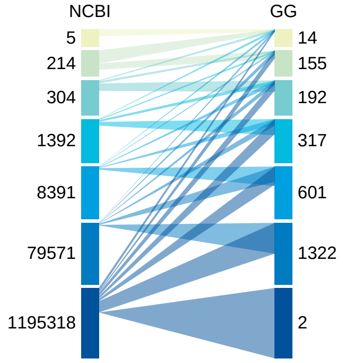

NCBI \ GG	R	D	P	C	O	F	G	S
R	1.00	0.00	0.00	0.00	0.00	0.00	0.00	0.00
D	0.60	0.40	0.00	0.00	0.00	0.00	0.00	0.00
P	0.27	0.50	0.23	0.00	0.00	0.00	0.00	0.00
C	0.70	0.03	0.06	0.21	0.00	0.00	0.00	0.00
O	0.85	0.01	0.02	0.04	0.08	0.00	0.00	0.00
F	0.94	0.00	0.01	0.01	0.01	0.03	0.00	0.00
G	0.96	0.00	0.00	0.01	0.01	0.02	0.01	0.00
S	0.65	0.05	0.02	0.05	0.03	0.03	0.16	0.00

Loose mapping

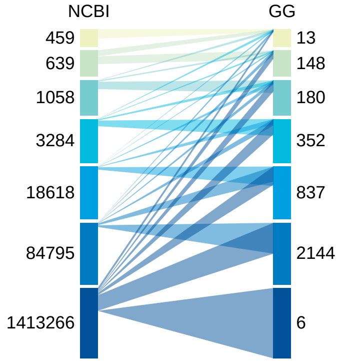

NCBI \ GG	R	D	P	C	O	F	G	S
R	1.00	0.00	0.00	0.00	0.00	0.00	0.00	0.00
D	0.47	0.53	0.00	0.00	0.00	0.00	0.00	0.00
P	0.48	0.23	0.29	0.00	0.00	0.00	0.00	0.00
C	0.75	0.01	0.03	0.21	0.00	0.00	0.00	0.00
O	0.84	0.00	0.01	0.02	0.13	0.00	0.00	0.00
F	0.94	0.00	0.00	0.00	0.01	0.04	0.00	0.00
G	0.93	0.00	0.00	0.00	0.01	0.03	0.03	0.00
S	0.68	0.05	0.01	0.01	0.01	0.03	0.22	0.00

Path comparison

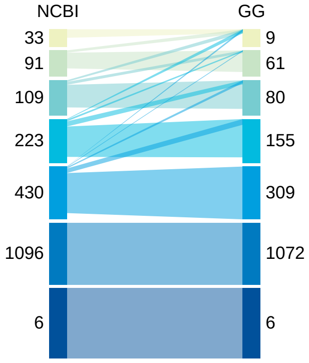

NCBI \ GG	R	D	P	C	O	F	G	S
R	1.00	0.00	0.00	0.00	0.00	0.00	0.00	0.00
D	0.52	0.48	0.00	0.00	0.00	0.00	0.00	0.00
P	0.32	0.09	0.59	0.00	0.00	0.00	0.00	0.00
C	0.23	0.05	0.08	0.64	0.00	0.00	0.00	0.00
O	0.14	0.01	0.04	0.12	0.69	0.00	0.00	0.00
F	0.12	0.00	0.01	0.02	0.09	0.76	0.00	0.00
G	0.00	0.00	0.00	0.00	0.00	0.00	1.00	0.00
S	0.00	0.00	0.00	0.00	0.00	0.00	0.00	1.00

Greengenes to OTT

Strict mapping

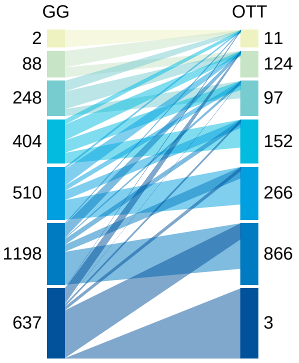

OTT (columns) vs. GG (rows)

GG	R	D	P	C	O	F	G	S
R	1.00	0.00	0.00	0.00	0.00	0.00	0.00	0.00
D	0.00	1.00	0.00	0.00	0.00	0.00	0.00	0.00
P	0.00	0.63	0.38	0.00	0.00	0.00	0.00	0.00
C	0.00	0.31	0.50	0.19	0.00	0.00	0.00	0.00
O	0.00	0.12	0.36	0.28	0.24	0.00	0.00	0.00
F	0.00	0.05	0.31	0.10	0.18	0.37	0.00	0.00
G	0.00	0.02	0.22	0.02	0.09	0.11	0.54	0.00
S	0.00	0.01	0.22	0.00	0.03	0.05	0.68	0.00

Loose mapping

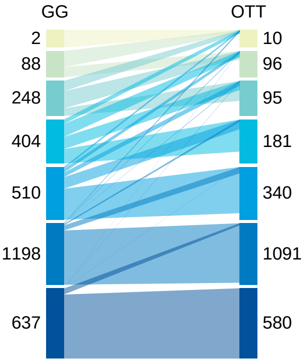

OTT (columns) vs. GG (rows)

GG	R	D	P	C	O	F	G	S
R	1.00	0.00	0.00	0.00	0.00	0.00	0.00	0.00
D	0.00	1.00	0.00	0.00	0.00	0.00	0.00	0.00
P	0.00	0.61	0.39	0.00	0.00	0.00	0.00	0.00
C	0.00	0.29	0.49	0.22	0.00	0.00	0.00	0.00
O	0.00	0.10	0.29	0.28	0.33	0.00	0.00	0.00
F	0.00	0.04	0.07	0.09	0.22	0.59	0.00	0.00
G	0.00	0.01	0.00	0.00	0.02	0.08	0.88	0.00
S	0.00	0.00	0.00	0.00	0.00	0.00	0.08	0.91

Path comparison

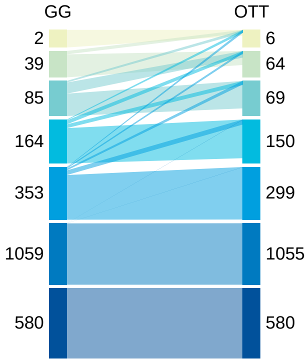

OTT (columns) vs. GG (rows)

GG	R	D	P	C	O	F	G	S
R	1.00	0.00	0.00	0.00	0.00	0.00	0.00	0.00
D	0.00	1.00	0.00	0.00	0.00	0.00	0.00	0.00
P	0.00	0.13	0.87	0.00	0.00	0.00	0.00	0.00
C	0.00	0.05	0.32	0.64	0.00	0.00	0.00	0.00
O	0.00	0.02	0.09	0.09	0.80	0.00	0.00	0.00
F	0.00	0.01	0.02	0.03	0.09	0.85	0.00	0.00
G	0.00	0.00	0.00	0.00	0.00	0.00	1.00	0.00
S	0.00	0.00	0.00	0.00	0.00	0.00	0.00	1.00

OTT to Greengenes

Strict mapping

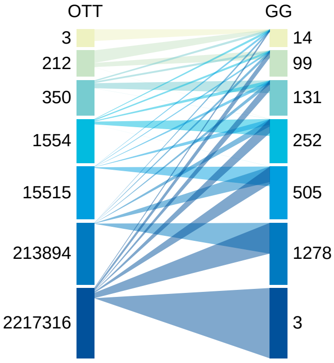

GG (columns) vs. OTT (rows)

OTT	R	D	P	C	O	F	G	S
R	1.00	0.00	0.00	0.00	0.00	0.00	0.00	0.00
D	0.33	0.67	0.00	0.00	0.00	0.00	0.00	0.00
P	0.36	0.48	0.16	0.00	0.00	0.00	0.00	0.00
C	0.77	0.04	0.05	0.14	0.00	0.00	0.00	0.00
O	0.88	0.01	0.03	0.02	0.06	0.00	0.00	0.00
F	0.97	0.00	0.01	0.00	0.00	0.01	0.00	0.00
G	0.99	0.00	0.00	0.00	0.00	0.01	0.00	0.00
S	0.85	0.01	0.03	0.00	0.00	0.02	0.08	0.00

Loose mapping

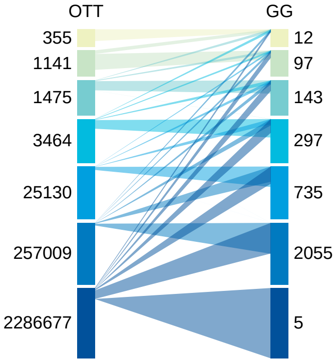

GG (columns) vs. OTT (rows)

OTT	R	D	P	C	O	F	G	S
R	1.00	0.00	0.00	0.00	0.00	0.00	0.00	0.00
D	0.33	0.67	0.00	0.00	0.00	0.00	0.00	0.00
P	0.26	0.14	0.60	0.00	0.00	0.00	0.00	0.00
C	0.71	0.01	0.02	0.26	0.00	0.00	0.00	0.00
O	0.78	0.00	0.01	0.01	0.20	0.00	0.00	0.00
F	0.93	0.00	0.00	0.00	0.00	0.06	0.00	0.00
G	0.96	0.00	0.00	0.00	0.00	0.01	0.03	0.00
S	0.84	0.01	0.00	0.00	0.00	0.02	0.12	0.00

Path comparison

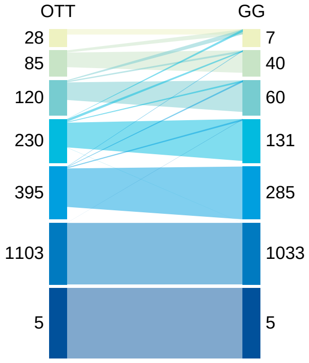

GG (columns) vs. OTT (rows)

OTT	R	D	P	C	O	F	G	S
R	1.00	0.00	0.00	0.00	0.00	0.00	0.00	0.00
D	0.71	0.29	0.00	0.00	0.00	0.00	0.00	0.00
P	0.36	0.08	0.55	0.00	0.00	0.00	0.00	0.00
C	0.44	0.03	0.03	0.50	0.00	0.00	0.00	0.00
O	0.35	0.00	0.06	0.02	0.56	0.00	0.00	0.00
F	0.24	0.00	0.01	0.01	0.02	0.73	0.00	0.00
G	0.00	0.00	0.00	0.00	0.00	0.00	1.00	0.00
S	0.00	0.00	0.00	0.00	0.00	0.00	0.00	1.00

NCBI to OTT

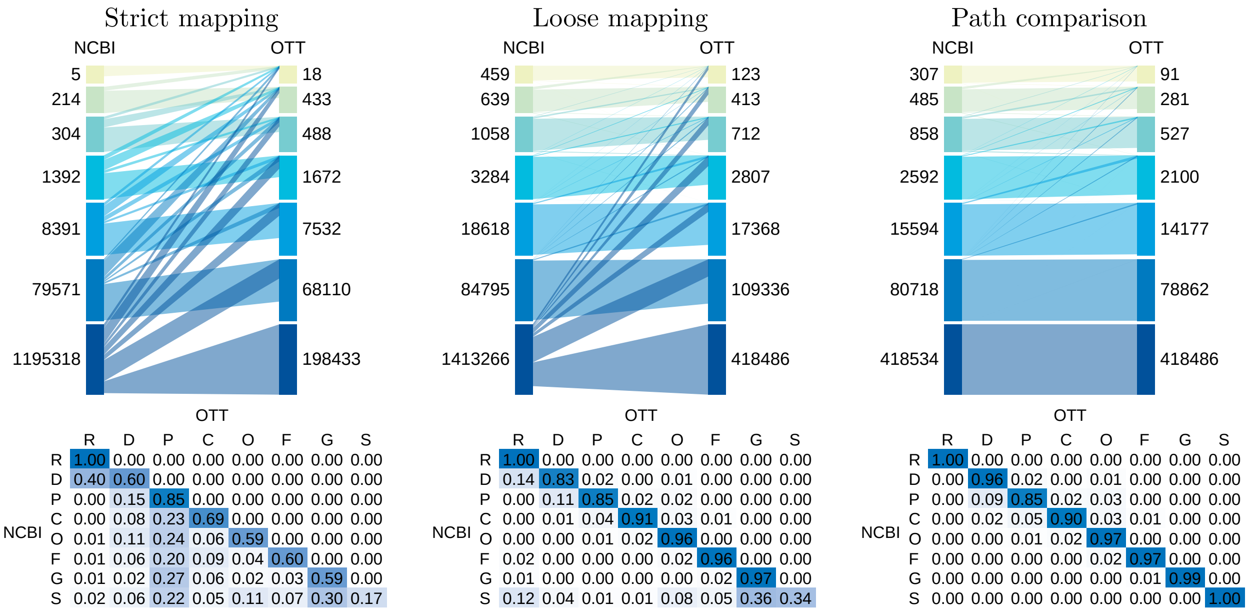

OTT to NCBI

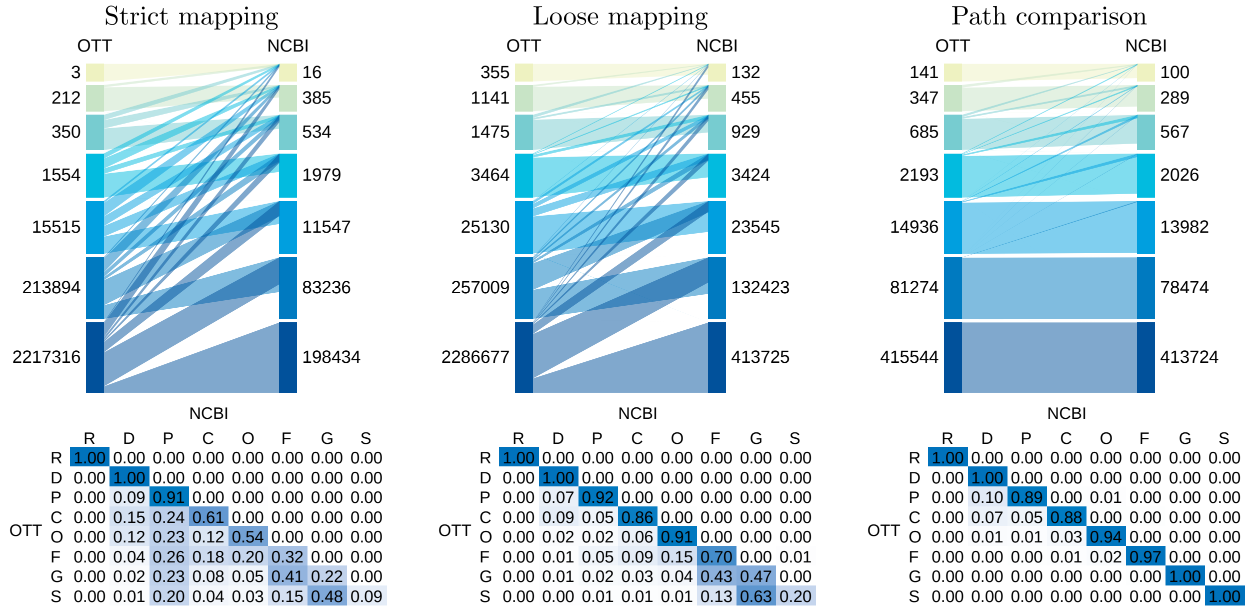

Part C: Comparisons limited to Bacteria and Archaea domains

	Strict	Loose	Path	Sparklines
RDP to SILVA	0.14	0.03	0.01	
Greengenes to SILVA	0.21	0.12	0.03	
NCBI to SILVA	0.18	0.08	0.02	
OTT to SILVA	0.28	0.13	0.00	
SILVA to RDP	0.21	0.10	0.01	
Greengenes to RDP	0.21	0.15	0.02	
NCBI to RDP	0.23	0.12	0.01	
OTT to RDP	0.30	0.19	0.00	
SILVA to Greengenes	0.24	0.16	0.02	
RDP to Greengenes	0.17	0.13	0.01	
NCBI to Greengenes	0.25	0.16	0.02	
OTT to Greengenes	0.32	0.21	0.01	
SILVA to NCBI	0.15	0.07	0.02	
RDP to NCBI	0.12	0.03	0.00	
Greengenes to NCBI	0.19	0.10	0.02	
OTT to NCBI	0.23	0.08	0.00	
SILVA to OTT	0.23	0.08	0.02	
RDP to OTT	0.19	0.04	0.01	
Greengenes to OTT	0.26	0.11	0.02	
NCBI to OTT	0.19	0.04	0.02	

Table S3: Mapping dissimilarities for all pars of taxonomies limited to Bacteria and Archaea domains. Sparklines indicate the magnitudes of strict, loose and path mapping dissimilarities for each pair of taxonomies and are provided as a quick overview of the table.

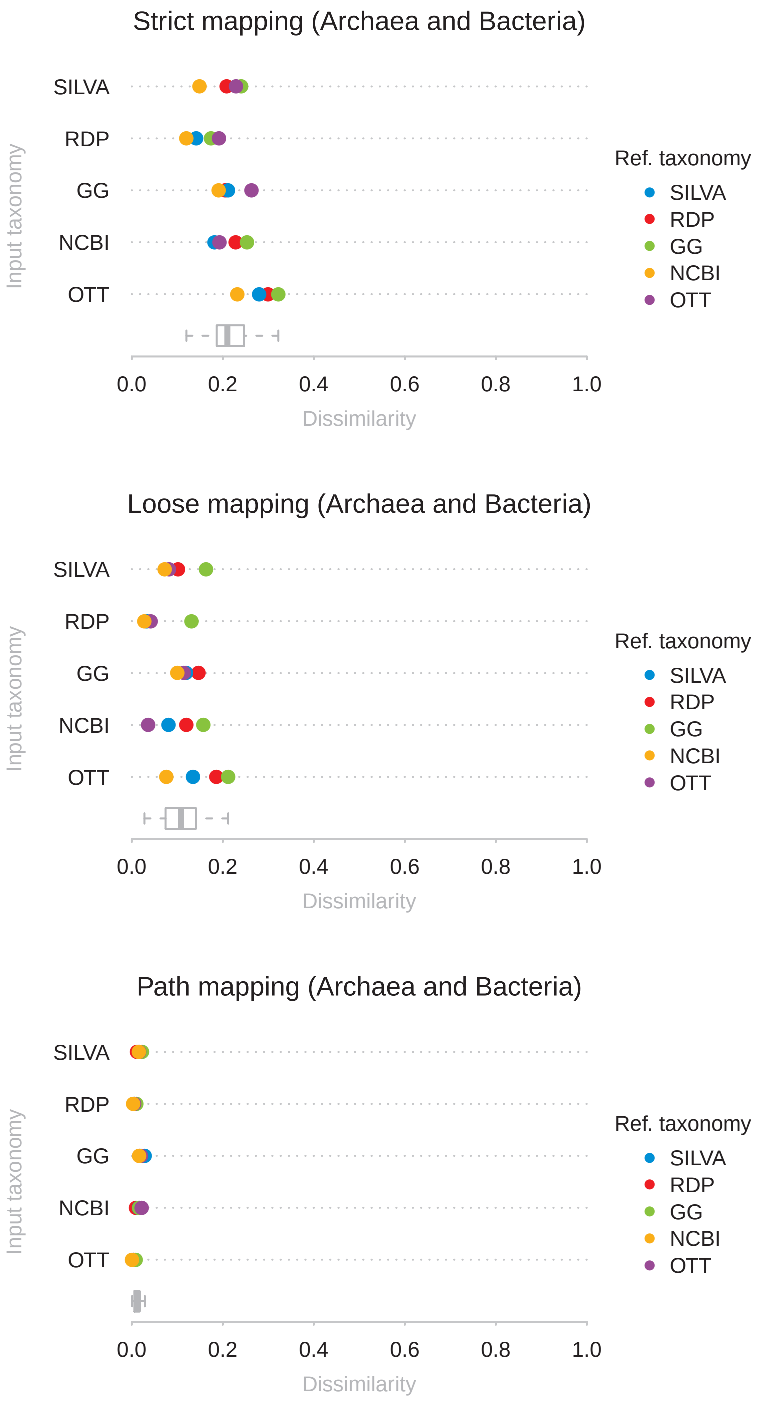

Figure 1: Distributions of mapping dissimilarities.
